# Gonadal dysfunction in women with diabetes mellitus

**DOI:** 10.1007/s12020-024-03729-z

**Published:** 2024-02-14

**Authors:** Maria Zaimi, Olympia Michalopoulou, Katerina Stefanaki, Paraskevi Kazakou, Vasiliki Vasileiou, Theodora Psaltopoulou, Dimitrios S. Karagiannakis, Stavroula A. Paschou

**Affiliations:** 1 School of Medicine, National and Kapodistrian University of Athens, Athens, Greece; 2 Department of Clinical Therapeutics, Endocrine Unit and Diabetes Centre, Alexandra Hospital, School of Medicine, National and Kapodistrian University of Athens, Athens, Greece; 3 Department of Endocrinology, Alexandra Hospital, Athens, Greece; 4 Academic Department of Gastroenterology, Laiko General Hospital, School of Medicine, National and Kapodistrian University of Athens, Athens, Greece

**Keywords:** Type 1 Diabetes Mellitus, Type 2 Diabetes Mellitus, Reproductive dysfunction, Menstrual disorders, Polycystic ovary syndrome (PCOS), Hypothalamus-pituitary-gonadal axis

## Abstract

It is well known that both type 1 and type 2 diabetes mellitus (DM) are related to increased risk for cardiovascular (CV) and chronic kidney disease (CKD). However, besides these prominently presented complications, DM has also been associated with reproductive dysfunctions. It seems that these disorders are met in up to 40% of women with DM and consist of delayed menarche, all types of menstrual disorders, such as amenorrhea, oligomenorrhea, menstrual irregularity, as well as menorrhagia, infertility, characteristics of polycystic ovary syndrome (PCOS) and early (or rarely late) menopause. In type 1 DM (T1DM), insulin treatment, although it has reduced the rates of insulinopenic-induced hypogonadotropic hypogonadism, an entity commonly presented in many women with the disease in the past decades, when it is used in excess it can also promote hyperandrogenism. Regarding type 2 DM (T2DM), insulin resistance (IR) and hyperinsulinemia have mainly been implicated in the pathogenesis of reproductive dysfunctions, as insulin can act as gonadotropin on the theca cells of the ovary and can lead to hyperandrogenism and inhibition of proper ovulation. This review aims to detail the reproductive dysfunctions associated with DM and provide scientific data to enlighten the underlying pathogenetic mechanisms.

## Introduction

Diabetes mellitus (DM) is a disorder with increasing prevalence worldwide. According to the International Diabetes Federation Diabetes Atlas, 537 million people have diabetes in 2021. By 2045, the number of people with diabetes is estimated to reach 783 million [1]. Additionally, in 2021, 6.7 million deaths were attributed to diabetes [1] by 3% between 2000 and 2019 [2]. The increased proportion of patients with DM creates a continuing challenge in the area of diabetes care and the management of its complications. Concerning the latter, cardiovascular (CV) diseases, strokes, chronic kidney diseases, retinopathy, and neuropathy are the most commonly developed; however, reproductive dysfunctions may occur as well [3]. As the number of women of reproductive age diagnosed with DM has increased, the prevalence of reproductive abnormalities due to DM will subsequently tend to be higher [3].

Codner et al. demonstrated that almost 40% of women with type 1 DM (T1DM) will present reproductive abnormalities, such as menstrual irregularity, hyperandrogenism, infertility, or menopause during their lifetime [4]. The onset of the above may occur early in the reproductive life of women with T1DM due to the complete lack of insulin. Insulin administration is the cornerstone of T1DM treatment, as it is a vital hormone. Insulin has been shown to improve the rhythm of menstrual cycles and prevent infertility [4]. However, it has been associated with new complications, such as hyperandrogenism and PCOS, in T1DM subjects [5, 6]. Albeit hyperandrogenism and PCOS accompany T1DM in premenopausal women at a high rate, it is essential to highlight the scarcity of evidence so far. Moreover, the pathophysiology behind this relationship is widely unknown [5].

Regarding type 2 DM (T2DM), approximately 462 million people were diagnosed worldwide in 2017. By 2030, the prevalence of T2DM is expected to increase by almost 7079 per 100,000 individuals globally [7]. Conversely to T1DM, T2DM is usually characterized by insulin resistance (IR) and hyperinsulinemia [8]. Gene factors and lifestyle modifications, such as obesity, smoking, high serum lipid levels, and absence of exercise, predispose to the development of T2DM [7]. Obesity and IR have been shown to increase the risk of menstrual irregularities, infertility, and the development of Polycystic Ovary Syndrome (PCOS) among women with T2DM [3].

It is essential to highlight that gonadal dysfunction and all the reproductive disorders discussed in this review may be influenced by the levels of circulating prolactin. Prolactin regulates a variety of functions and contributes to the promotion of metabolic homeostasis [9]. Large clinical studies have recently shown that reducing circulating prolactin levels is associated with metabolic disease and represents a risk factor for T2DM [9, 10].

This review aims to provide recent scientific data regarding the pathogenesis of reproductive dysfunctions in women with T1DM and T2DM, along with clinical features. As the pathophysiological mechanisms underlying reproductive disorders differ between the two types of DM, they are discussed separately.

To comprehensively address these issues, we conducted searches on PubMed and the Cochrane Library, utilizing the key terms of “gonadal dysfunction,” “menstrual disorders,” or “reproductive dysfunction”, AND “type 1 diabetes”, “type 2 diabetes”, or “diabetes”. The search aimed to identify randomized control trials (RCTs), retrospective studies, and systematic reviews and meta-analyses. There was separate research on “PCOS” AND type 1 diabetes”, “type 2 diabetes”, or “diabetes,”

## Type 1 diabetes and reproductive function

### Hypothalamic-pituitary-gonadal axis

A synergistic action of many hormones regulates the hypothalamic-pituitary-gonadal (HPG) axis. Insulin is one of them and has been shown to stimulate the secretion of gonadotropin-releasing hormone (GnRH) [3]. Consequently, insulin deficiency in women with T1DM may lead to central functional hypogonadotropic hypogonadism. This correlation was first described in the 1980s in patients with T1DM and poor metabolic control, whose estradiol, FSH, and LH levels were excessively low [11]. Subsequently, several animal studies investigated the underlying pathophysiological mechanism of hypogonadism in diabetic mice. They demonstrated that those with uncontrolled hyperglycemia reached a catabolic state characterized by low leptin levels and decreased adipose tissue. Low insulin and low leptin were found, in turn, to inhibit the expression of kisspeptin in the central nervous system [3]. Kisspeptin has been shown to be a significant stimulator of GnRH production, and its deficiency leads to functional hypogonadism [12]. In addition, Codner et al. had previously indicated chronic glucotoxicity on GnRH hypothalamic neurons as another plausible mechanism responsible for blunted GnRH excretion [4]. The investigators confirmed the central-hypothalamic origin of hypogonadism, as an exogenous infusion of GnRH in T1DM patients was followed by a normal pituitary response [4].

### Ovarian function

In T1DM, chronic excessive insulin administration may provoke weight gain, which seems to correlate with an increased risk of hyperandrogenemia and the development of PCOS [4]. In normal subjects with intact pancreatic function, excreted insulin inserts the liver via the portal vein for a first intra-hepatic clearance; in cases of subcutaneous insulin infusion, such as in T1DM, the liver is bypassed, and high levels of insulin come through systemic circulation. As a result, an excess amount of insulin binds to ovarian IGF-1 receptors and stimulates the production of androgens from theca cells in prepubescent girls [3]. Interestingly, an increased number of small follicles is associated with increased anti-mullerian hormone (AMH) levels, as observed in prepubescent girls with T1DM [4].

Besides the negative direct role of insulin infusion, a poorly controlled T1DM, defined by hyperglycemia and subsequent glycation of ovarian receptors, disrupts ovarian folliculogenesis, leading to enhanced follicular apoptosis and early menopause [4].

## Menstrual disorders in T1DM

### Delayed menarche

Adolescents diagnosed with T1DM before the age of 10 have higher rates of 1-year-delayed menarche compared to those diagnosed after that age [13–15]. Schweiger and colleagues confirmed that delayed menarche occurred with significantly higher rates in adolescents diagnosed with T1DM before the menarche compared to healthy adolescents [16]. These findings are pretty significant, as early assessment of delayed menarche is not merely associated with a higher risk of menstrual disorders but also with a higher risk for cardiovascular and diabetic microvascular complications, including nephropathy and retinopathy [4, 17]. The advances in the care of T1DM during the last decades with multiple insulin injections daily or ‘pumps’ optimized metabolic control, diminished hypogonadism, and pubertal delay [3]. Findings from a Japanese cohort of children and adolescents with T1DM concluded that glycemic control tended to improve among the 1995 to 2008 cohorts, and the pubertal increase in HbA1c levels among female patients tended to decrease among the sequential cohorts. As a result, the increased use of basal-bolus insulin regimens seems to improve glycemic control and diminish the frequency of hypoglycemic events [18]. Another Japanese study concluded that HbA1c levels at 10, 11, and 12 years of age were lower than 8.0%, and the number of patients using continuous subcutaneous insulin infusion (CSII) increased significantly at the age of 10, 11, and 12 during the last years. Consequently, better glycemic control may positively affect the onset of menarche [19].

### Menstrual irregularities

Menstrual disorders occur at higher rates in adolescents with T1DM compared to healthy controls [4, 13]. Hence, it seems that T1DM patients have a six times higher risk for oligomenorrhoea and increased duration of menstrual cycles compared to healthy women [3]. Many studies have clarified that poor metabolic control, defined by a 1% elevation in HbA1c, significantly increases the risk of oligomenorrhea and prolongs the menstrual cycle by 5.1 days, on average [4, 13, 20]. Though optimal metabolic control usually reverses the above menstrual disorders, possible persistence should not be excluded [13]. Probably, not only glycemic status but also hyperandrogenism seem to play some crucial role. Indeed, it has been verified that adolescents with menstrual abnormalities and T1DM often have clinical and hormonal characteristics consistent with PCOS (i.e., polycystic ovarian appearance, low levels of SHBG, and an elevated LH/FSH ratio) [21, 22]. Interestingly, a French study confirmed the association between androgen levels and oligomenorrhea, as oligomenorrheic adolescents with T1DM had higher androgen levels than adolescents with T1DM and regular menstrual cycles [23]. Moreover, a recent Greek study of 53 young women with T1DM demonstrated that increased androgen bioavailability due to decreased SHBG concentrations may result in delayed menarche, oligomenorrhea, and clinical hyperandrogenism [24].

## Type 2 diabetes and reproductive function

### Hypothalamic-pituitary-gonadal axis

T2DM is predominantly characterized by IR and hyperinsulinemia, which has already been mentioned to exacerbate GnRH secretion, resulting in increased levels of LH/FSH. These, in turn, stimulate ovarian folliculogenesis, induce ovarian stroma hyperplasia, and increase androgen production [3]. Besides insulin, hormones excreted by adipose tissue are also implicated. Hence, leptin stimulates GnRH production through kisspeptin, adiponectin, and ghrelin inhibit GnRH. In contrast, Neuropeptide Y (NPY) either stimulates or inhibits GnRH secretion depending on the patient’s metabolic status (i.e., negative energy balance promotes inhibition of GnRH secretion). Nevertheless, the exact role of these hormones on HPG function or the factors that regulate their circulating levels have not yet been clarified [25]. Nevertheless, leptin resistance plays an essential role in the pathogenesis of hypothalamic hypogonadism. Leptin resistance is related to human obesity owing to downregulated receptors or post-receptor implications [26]. A study in female transgenic skinny mice overexpressing leptin suggested that chronic hyperleptinemia, as found in obesity, may downregulate leptin signaling in the hypothalamus, resulting in decreased hypothalamic function [27]. In addition, a comparative study by Balasubramanian et al. in rats concluded that hypothalamic leptin resistance may indirectly result in reduced hypothalamic kisspeptin expression and a concomitant decrease in hypothalamic GnRH and LH secretion [28].

It is well known that obesity is a crucial risk factor for T2DM, further complicating its management [29]. However, obesity may result in gonadal dysfunction and functional hypogonadotropic hypogonadism [30]. A recent journal article suggested the concept of non-PCOS ‘female obesity-related secondary hypogonadism’ (FOSH), an analog to the well-established male obesity-related secondary hypogonadism (MOSH) [26]. The pathophysiology behind FOSH is multifactorial. Obesity is associated with low LH levels, whereas in women with obesity, lower LH levels may result from increased clearance of endogenous LH and diminished pituitary response to GnRH. Obese women may have increased androgen levels, and an intense increase may lower LH levels. Another pathophysiologic mechanism constitutes the increase of inflammatory markers in obesity, which may result in decreased LH levels and hypogonadism. Nevertheless, further research is necessary to confirm the pathogenesis of FOSH and increase our knowledge regarding this aspect [26].

Lastly, recent studies have indicated that patients with T2DM have a higher prevalence of “hidden hypercortisolism”. This condition does not present with the usual findings of Cushing’s syndrome but rather with camouflaged manifestations, such as hypertension, bone fragility, and T2DM. As a result, high cortisol levels in T2DM may have an inhibitory action in the Hypothalamic-Pituitary-Ovarian axis, contributing to the aforementioned reproductive disorders [31].

### Ovarian function

T2DM disrupts normal ovarian function since IR and hyperinsulinemia trigger the overproduction of IGF-1 and overexpression of hybrid insulin/IGF-1 receptors [3]. As a result, ovarian granulosa cells are stimulated, abnormal folliculogenesis is induced, and enlarged ovaries of multiple small follicles (polycystic ovarian morphology) are developed. The IGF receptors’ overactivity leads to higher production of ovarian androgens, as insulin maintains its action on the ovary, resulting in clinical hyperandrogenism in women with T2DM. Additionally, high insulin concentrations, acting as gonadotropin on the theca cells of the ovaries, inhibit the recruitment of one dominant follicle, leading to menstrual disturbances and anovulation [3].

## Menstrual disorders in T2DM

### Early menarche

The risk for T2DM is significantly higher among women with early menarche [32]. A systematic review and meta-analysis of 10 studies concluded that women with early menarche had a higher risk of T2DM compared to women with menarche in older age. Genetic factors, higher estrogen levels, lower SHBG levels, and obesity before puberty have been implicated [32]. A recent meta-analysis confirmed the above results, identifying a 1.3 times higher risk for T2DM in patients with early menarche [33]. In accordance with these findings, a cross-sectional study of 23.138 women showed that later menarche (≥ 18 years) was associated with a 17.7% lower risk of T2DM (95% CI: 0.712–0.951, *p* = 0.008) [34].

Inversely, any potential impact of T2DM on the time of menarche is challenging to investigate due to the extremely low prevalence of T2DM in prepubescent girls [3].

### Menstrual irregularities

Women with youth-onset T2DM have higher rates of menstrual irregularities, such as oligomenorrhea, compared to healthy adolescents [3]. In a Korean study, oligomenorrhea in women with T2DM during their 20 s was found to be two times more frequent compared to controls (16.1% vs. 8.5%, *p* = 0.03). Interestingly, it was shown that the development of oligomenorrhea may precede the diagnosis of T2DM [35].

Menstrual abnormalities and T2DM share common risk factors, including obesity, visceral adiposity, IR, and hyperinsulinemia [3, 35]. Obesity drives the peripheral aromatization of androgens to estrogens in adipose tissue, increasing the risk for menstrual irregularities [35, 36]. Moreover, increased BMI is closely linked to anovulation and menstrual cycle abnormalities. On the other hand, hyperinsulinemia induces the production of androgens in ovarian theca cells and decreases SHBG levels, resulting in high free androgen levels and oligomenorrhea [35]

## Polycystic Ovary Syndrome (PCOS)

PCOS is the most frequent endocrinopathy in women of reproductive age. According to the 2018 International Guidelines, PCOS may be diagnosed if any two of the following are present: (a) clinical or biochemical hyperandrogenism, (b) evidence of oligo-anovulation (cycles >35 days apart or <8 menses a year), (c) polycystic appearing-ovarian morphology on ultrasound, with the exclusion of other relevant disorders [37–39].

### T1DM and PCOS

Data from a previous meta-analysis indicated that the prevalence of PCOS in women with T1DM was close to 24%, higher than in the general population. Moreover, women with T1DM had hyperandrogenemia in 25%, hirsutism in 25%, menstrual dysfunction in 24%, and polycystic ovary morphology in 33% [5]. Another meta-analysis recently confirmed these findings, which yielded 19 studies and included 1042 women with T1DM [40].

The pathophysiologic association between T1DM and PCOS remains unclear, and the evidence regarding this aspect is limited [5]. It has been postulated that insulin treatment in T1DM may trigger PCOS in women predisposed to this condition [5, 41]. The administration of exogenous insulin results in prolonged exposure of the ovaries to high insulin concentrations and may increase androgen production among women with a predisposition [6]. These findings are similar to the ones that characterize PCOS. However, there are differences in the biochemical profile of women with T1DM compared to those with PCOS. Mainly, SHBG levels are low in PCOS, compared to T1DM but not in T1DM [4, 5], and free androgen concentrations are lower in women with T1DM compared to non-diabetic women with PCOS [5]. Insulin is considered an essential regulator of SHBG secretion and metabolism. Interestingly, the SHBG concentrations in women with T1DM and PCOS are not reduced, possibly due to subcutaneous administration of insulin [42]. Precisely, hyperinsulinemia, which commonly presents in PCOS due to IR, downregulates the SHBG gene transcription and, therefore, the SHBG production. However, SHBG levels are mainly regulated by insulin concentration at the portal vein. In T1DM, insulin is administered subcutaneously to the systemic circulation, and as a consequence, it results in lower portal levels of insulin, as well as lower SHBG levels compared to PCOS [4, 43]. Furthermore, free androgen concentrations are lower in women with T1DM compared to non-diabetic ones with PCOS [5].

### T2DM and PCOS

#### Development of PCOS in women with T2DM

PCOS and T2DM seem to share a bidirectional relationship. According to a recent meta-analysis, the worldwide prevalence of PCOS in women with T2DM of all ages is approximately 21%. The percentage of PCOS was higher in women with T2DM diagnosed at the childbearing age than in adolescents [44]. Similar results were shown in the meta-analysis of Cioana et al., where the prevalence of PCOS in adolescent women aged at the time of T2DM diagnosis between 12.9 and 16.1 years old was 19.6% [45].

Recent studies have shown that women with PCOS are at increased risk of developing T2DM, IGT, and metabolic syndrome (MS) compared to women without [46, 47]. Notably, in the analysis of Moran et al., the prevalence of T2DM among women with PCOS was more significant compared to women without [OR 4.43 (4.06, 4.82) on fixed-effect analysis and OR 3.16 (95% CI 1.87–5.32) on random-effects analysis] [46]. Results from another meta-analysis showed similar findings, underpinning a higher rate of T2DM among women with PCOS compared to women without (RR: 3.00, 95% CI 2.56–3.51) [48].

Obesity is undoubtedly a contributing factor in developing T2DM. However, other factors seem also to be implicated, as obese PCOS women have been found to have higher rates of T2DM compared to obese non-PCOS subjects (RR: 3.24) [49]. Similarly, it has been shown that women with PCOS have a 27% reduction in insulin sensitivity irrespective of BMI, with obesity just worsening IR by 15% [49, 50]. Interestingly, while indicating a positive association between higher BMI, higher testosterone levels, and lower SHBG and the development of T2DM, a two-sample Mendelian randomization study did not find an association between the genetic risk of PCOS and that of T2DM [51].

## Fecundity

### Type 1 diabetes mellitus

There is scarce data addressing fertility issues in T1DM. A small number of studies have demonstrated a correlation between T1DM and infertility. Α Swedish population-based cohort study showed that women with T1DM, especially those with T1DM-related complications, had a lower fertility ratio. Moreover, a retrospective Taiwanese survey confirmed that women of reproductive age with T1DM had a lower rate of live births (IRR 0.67) compared to non-diabetic controls. The lower live-birth ratio was attributed to gestation and obstetrical complications rather than a higher abortion rate [52]. Similarly, another group of investigators indicated a 24% reduction in the Fecundability Odds Ratio (FOR) of women with T1DM in comparison to non-diabetic women [53]. Noteworthy, optimal glycemic control seems to be associated with improvement in fertility, as higher ovulatory rates have been found in healthy- than poor-controlled women (51.3% vs. 29.4% respectively) [54].

The etiopathogenesis of subfertility among women with T1DM is complex and multifactorial. Decreased fecundability may be explained by diabetes-related complications or sexual dysfunction (SD) [55, 56]. It has already been mentioned the role of insulin deficiency and low leptin levels in the development of hypogonadotropic hypogonadism and menstrual abnormalities. Subfertility is expressed by lower levels of anti-Müllerian hormone (AMH) in T1DM compared to healthy controls (weighted mean difference (WMD) 0.70 ng/ml, 95% CI:1.05–0.34 ng/ml, *p* = 0.0001) [57, 58]. Since AMH correlates with ovarian reserves and the number of follicles, its reduction increases the risk of early menopause in these patients [4].

### Type 2 diabetes mellitus

Women with T2DM are at increased risk of infertility [3]. The FOR among women with T2DM was 36% lower compared to non-diabetic women in the study of Whitworth et al. (2011) [53]. The risk of infertility increases with poor glycemic control and the presence of diabetic complications as ovarian reserves deteriorate and antral follicle count declines [59].

### Circadian clocks and fertility

The stressful modern lifestyle is associated with hormonal changes and consequent development of infertility-related conditions due to disrupted hypothalamic–pituitary–gonadal axis [60]. In men and women, fertility is regulated by clock genes [61]. The disrupted expression of clock genes affects infertility via decreased levels of sex hormones, failure of embryo implantation, and decreased newborn size among both mouse models and shift-working women [60]. Core and clock-controlled genes constitute the circadian clock mechanism. The circadian clock directs metabolic variations during the day; conversely, the circadian excretion of insulin regulates the expression of clock genes [62]. It is essential to highlight that circadian rhythm plays a central role in regulating metabolic function. The pancreas releases insulin to evoke glucose uptake after food ingestion. However, insulin continues to be released following the circadian clock in experimental fasting in humans. The disturbed circadian clock, i.e., in simulated shift-work conditions, alters circulating leptin, glucose, and insulin levels in prediabetic conditions. On the other hand, metabolic signals and insulin levels control the Circadian Function [63].

## Sexual dysfunction

### Sexual dysfunction in women with T1DM

SD is a common diabetic complication in people with either T1DM or T2DM. Whereas SD affects both genders, it is observed among women at a lower rate [64]. The pathophysiology behind SD in women is multifactorial, including hormonal, neuropathic, and psychosocial factors [65].

Low libido, dyspareunia, decreased lubrication, deficient arousal, and inability to orgasm constitute the most commonly reported features of SD [66–68]. A qualitative Norwegian study confirms the findings above, as reduced sexual desire, vaginal dryness, and pain during sexual activity were the most commonly described disorders in women with T1DM [68].

High glucose levels may decrease the hydration of vaginal mucous membranes, resulting in reduced lubrication and dyspareunia. Moreover, dyspareunia risk is increased owing to the higher risk of vaginal infections. Vascular damage and consequent neuropathy may decrease genital blood flow and compromise genital arousal. Importantly, psychosocial factors, the acceptance and living with a chronic disease, in addition to depression, may lead to SD. The pathophysiology above applies to SD in both types of DM [69].

Data show that the prevalence of SD is higher among women with T1DM compared with women with T2DM or without diabetes [66, 67]. Hashim et al., in their review, observed the high frequency of SD among women with T1DM and the three times higher risk of SD compared with healthy women.

Women described that T1DM implicated their mental health as a crucial stress factor and negatively affected their sexual contact with their partner [68]. A qualitative study concluded that women with T1DM were negatively influenced by additional factors associated with DM regarding their sexual function, including visible tissue damage due to insulin injection, fear of hypoglycemia during sexual contact, and wearable diabetes technologies [70]. On the other hand, SD may further lower their life quality and hinder their relationships, increasing anxiety and depression [71]. Another study from Norway found a higher prevalence of SD in women with T1DM (50.3%) compared with healthy women (35.0%) [72].

However, there is a lack of attention and knowledge regarding FSD, possibly owing to social and cultural factors and consequent unproductive dialog with healthcare professionals regarding this problem [71]. The high prevalence of SD and its negative effect on life and intimacy necessitates its appropriate treatment after the successful diagnosis of SD in women [71].

### Sexual dysfunction in women with T2DM

SD in women, as previously mentioned, includes low sexual desire, disorders of genital arousal, orgasm disorder, pain, and difficult intercourse. Sexual drive, libido, vaginal lubrication, and overall satisfaction were lower in women with T2DM compared with the control group based on the results of a study. These characteristics of SD were negatively correlated with the duration of diabetes and age, whereas they were not significantly related to BMI, glycemic control, education, or professional situation [73]. These results agree with the findings of another study, in which the Female Sexual Function scores in arousal, pain, orgasm, and overall satisfaction were also lower in the diabetic women and negatively correlated with the age of the women. Moreover, women with T2DM attempted sexual contact less frequently than healthy women, whereas no significant relationship between the FSF score and BMI, BP, and glycemic control was observed [74].

A recent systematic review and meta-analysis of ten studies, including 572 females with T2DM, found a pooled prevalence of SD of 52%. This study concluded that age >45 years old and or menopause and the administration of antihypertensives were related to SD [75]. Another case-control study suggested that the high rate of dysfunction regarding desire and arousal sensation may be associated with the higher mean age, higher BMI, vascular abnormalities, and the psychological burden of chronic disease. Additionally, the pain was more frequently observed among the control group than the case group, owing to better sensation [76]. Moreover, women with T2DM had poor mental stability at a higher frequency compared with healthy women associated with SD [77].

## Menopause

### Type 1 diabetes mellitus

Premature ovarian insufficiency and early menopause are observed more frequently in women with T1DM compared to healthy adults [3]. The Familial Autoimmune and Diabetes Study showed that women with T1DM had an earlier age of menopause (41.6 years) compared to their healthy sisters (49.9 years) or unrelated controls (48.0 years) [78]. Women developing T1DM before menarche exhibited delayed menarche (0.5-y delay, *P* = 0.002), earlier natural menopause (−2.0 y, *P* < 0.0001), and shorter reproductive period by 2.5 years compared to non-diabetic women (*P* < 0.0001) [79].

Nevertheless, Sjöberg et al. did not observe significantly different percentages of earlier menopause in diabetic women compared to the general population in Finland. In the case of early menopause, this was attributed to the microvascular complications of T1DM [80].

The early menopause and ovarian aging in T1DM have been associated with two pathogenetic mechanisms: autoantibodies against ovarian tissue and poor glycemic control, which both defect ovarian function and lead to early depletion of the ovarian follicles [3, 81]. This is defined by an early decline in AMH and inhibin-B serum concentrations [81, 82].

### Type 2 diabetes mellitus

#### Earlier menopause in women with T2DM

T2DM affects the onset of menopause. Women with T2DM had a three times higher risk of menopause before the age of 45 years compared to healthy women, according to a Latin American study [83]. Early onset T2DM may result in ovarian aging and consequently in earlier menopause attributed to microvascular ovarian derangements [81]. However, the increased premenopausal adipose tissue and weight among women with T2DM results in increased production of estrogens by androgen precursors in the adrenal glands and ovary, delaying menopause [81].

#### Development of T2DM after menopause

Menopause per se seems to predispose to the development of T2DM, as decreased estrogen levels affect the production and action of insulin negatively [81]. The Study of Women’s Health Across the Nation showed that low serum estradiol correlates with a higher risk of DM, and the risk increases among women with low estradiol levels across the transition to menopause [84–99].

## Conclusions and future perspectives

**Fig. 1:**
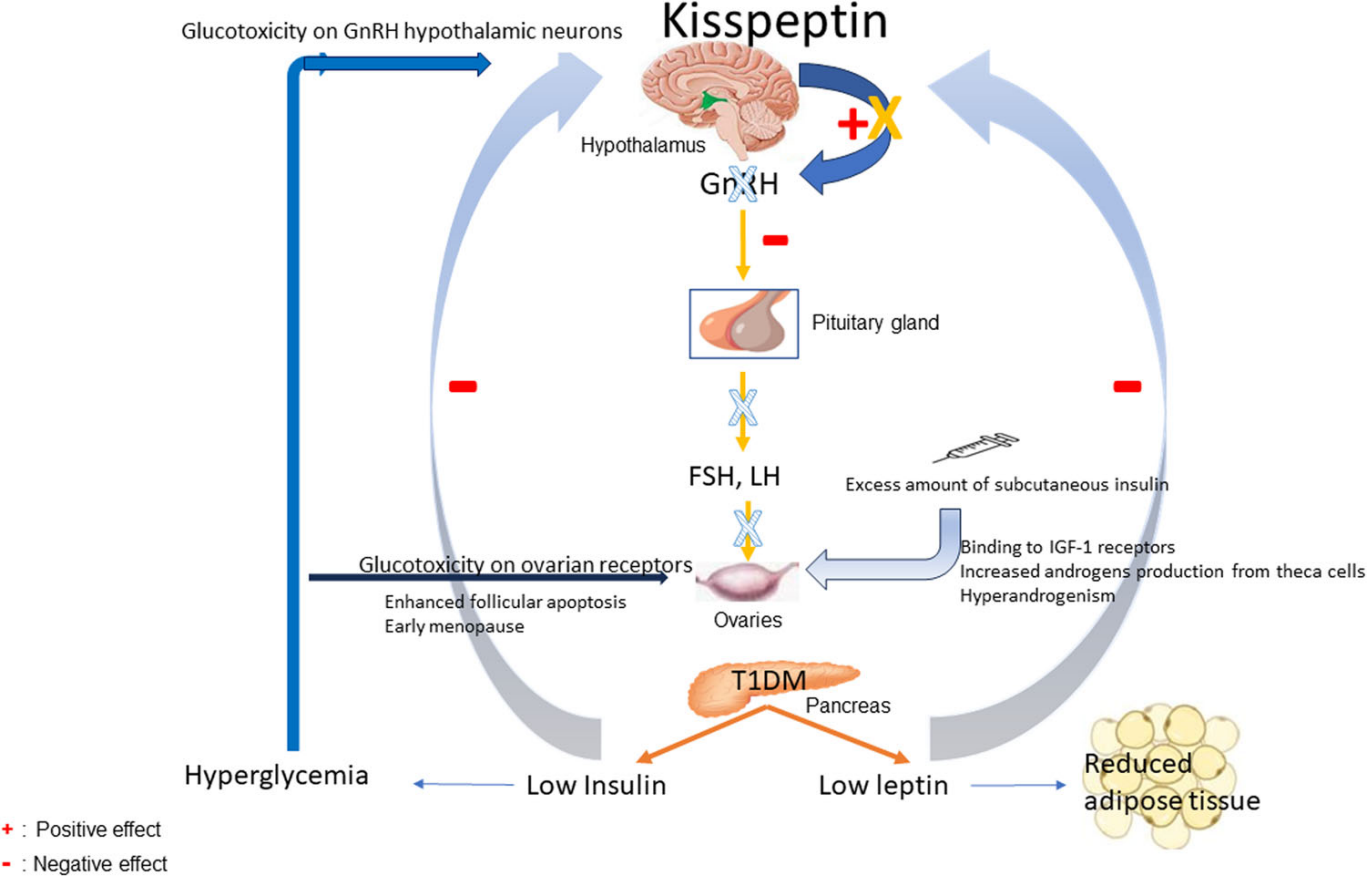
Pathophysiological mechanisms contributing to reproductive dysfunctions in T1DM

**Fig. 2:**
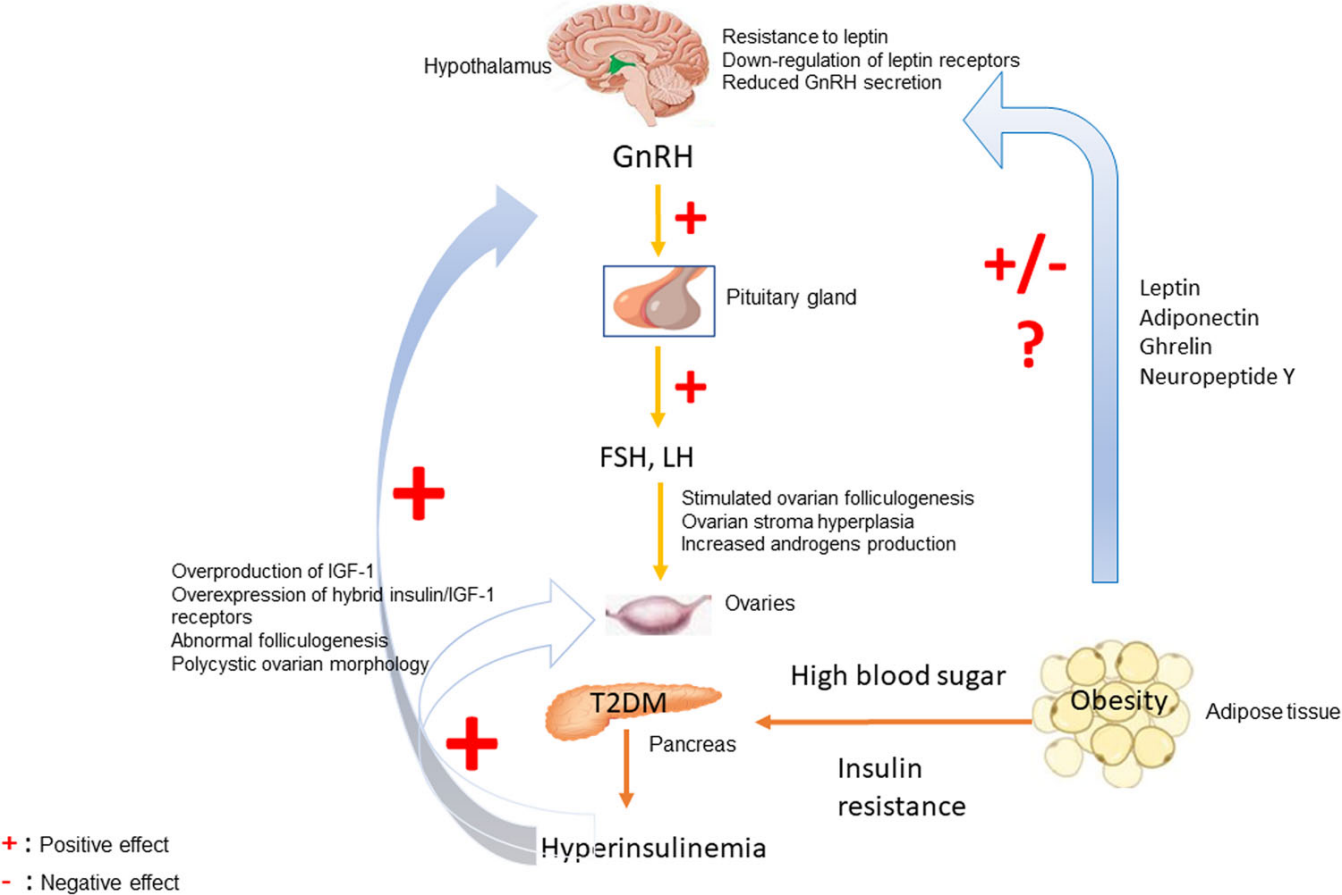
Pathophysiological mechanisms underlying reproductive abnormalities in T2DM

Women with either T1DM or T2DM are at increased risk of developing reproductive abnormalities throughout their lifetime compared to healthy women [Table 1]. T1DM is associated with 1-year-delayed menarche when the diagnosis of T1DM occurs before the age of 10, with six times higher risk for oligomenorrhea compared to healthy women, with increased levels of androgens and hormonal characteristics similar to PCOS [Fig. 1]. Additionally, infertility, subfertility, early ovarian aging, and menopause due to autoantibodies against ovaries and poor glycemic control are observed in women with T1DM. On the other hand, T2DM is associated with early menarche and oligomenorrhea due to the hormonal changes provoked by obesity [Fig. 2]. Moreover, women with T2DM are at increased risk of infertility, which is exacerbated by poor glycemic control. Additionally, increased levels of androgens in T2DM predispose to the development of PCOS, while PCOS through obesity and IR may inversely lead to T2DM. Similarly, a bidirectional relationship between T2DM and early menopause also exists. In both types of diabetes, preconception and pregnancy care should be promoted and applied to prevent maternal and fetal complications. Nonetheless, while reproductive dysfunctions presented in diabetic patients have been sufficiently recognized, they have been less studied in comparison to other well-known complications, such as cardiovascular diseases, nephropathy, and diabetic retinopathy.

**Table 1:** Reproductive dysfunction in diabetes

Diabetes	Reproductive Function	Reproductive Abnormality	Pathophysiologic mechanism
T1DM	Menstrual cycle	Delayed menarche	↓ or absent insulin→ ↓ kisspeptin → ↓ GnRH [3,4]
		Menstrual irregularities, Oligo-, amenorrhea, ↓duration of menstrual cycle	↓ SHBG→ ↑ androgens → menstrual disorders [17,20,18]
	Ovarian function	Follicular stimulation, Androgen production	Insulin → insulin and IGF-1 receptors activation in ovaries → ↑ small follicles or PCOS in predisposed women [3,4,5,31]
	Fertility	Reduced fertility	↓ or absent insulin→ ↓ kisseptin → ↓ GnRH → hypogonadotropic hypogonadism [3,4]
	Menopause	Earlier menopause	Autoantibodies + poor glycemic control→ destruction of ovarian tissue + ↓ ovarian follicles [3,51]
T2DM	Menstrual cycle	Early menarche	Genetic factors, ↑ estrogen, ↓ SHBG, obesity before puberty→ early menarche→ ↑ risk of T2DM [22]
		Menstrual irregularities Oligomenorrhea	Obesity + ↑ insulin + insulin resistance → ↑ androgen → menstrual disorders [25,26]
	Ovarian function	PCOS in T2DM	obesity→ ↑insulin + insulin resistance→ PCOS. Further studies needed [33]
		T2DM in PCOS	↑ insulin + insulin resistance → stimulation of granulosa cells→ large ovaries with multiple small follicles [3]
	Fertility	Reduced fertility	↑ insulin + insulin resistance→ inhibition of dominant follicle → anovulation [3]
	Menopause	Earlier menopause in T2DM	Early- onset T2DM → acceleration of ovarian ageing [51]
		T2DM after menopause	Menopause → ↓ estrogen → ↓ ERα activation by insulin + ↓ insulin secretion → ↓ insulin and insulin resistance [51]

The table describes the reproductive abnormalities that accompany women with diabetes. T1DM and T2DM are presented separately. The reproductive functions that are affected on each type of diabetes are divided into 4 categories: menstrual cycle, ovarian function, fertility and menopause. Thereafter, each reproductive dysfunction is described, and the suggested pathophysiological mechanism is analyzed.

↑: increase, ↓: decrease, →: results into, *T1DM* Type 1 Diabetes Mellitus, *T2DM* Type 2 Diabetes Mellitus, *PCOS* polycystic ovary syndrome, *GnRH* Gonadotropin-releasing hormone, *SHBG* sex Hormone Binding Globulin, *ERα* Estrogen receptor alpha.
